# Expanding the genetic toolbox for *Cutaneotrichosporon oleaginosus* employing newly identified promoters and a novel antibiotic resistance marker

**DOI:** 10.1186/s12896-023-00812-7

**Published:** 2023-09-18

**Authors:** Nikolaus I. Stellner, Zora S. Rerop, Norbert Mehlmer, Mahmoud Masri, Marion Ringel, Thomas B. Brück

**Affiliations:** 1 TUM School of Natural Sciences, Department of Chemistry, Werner Siemens-Chair for Synthetic Biotechnology, Technical University of Munich, Lichtenbergstr. 4, 85748 Garching, Germany; 2 TUM CREATE Ltd, 1 Create Way, #10-02 CREATE Tower, Singapore, 138602 Singapore

**Keywords:** *Cutaneotrichosporon oleaginosus*, Promoter, Dominant marker, Oleaginous yeast, Antibiotic resistance, Aminoglycoside 3’-phosphotransferase, N-acetyl transferase

## Abstract

**Background:**

*Cutaneotrichosporon oleaginosus* is an oleaginous yeast that can produce up to 80% lipid per dry weight. Its high capacity for the biosynthesis of single cell oil makes it highly interesting for the production of engineered lipids or oleochemicals for industrial applications. However, the genetic toolbox for metabolic engineering of this non-conventional yeast has not yet been systematically expanded. Only three long endogenous promoter sequences have been used for heterologous gene expression, further three dominant and one auxotrophic marker have been established.

**Results:**

In this study, the structure of putative endogenous promoter sequences was analyzed based on more than 280 highly expressed genes. The identified motifs of regulatory elements and translational initiation sites were used to annotate the four endogenous putative promoter sequences D9FADp, UBIp, PPIp, and 60Sp. The promoter sequences were tested in a construct regulating the known dominant marker hygromycin B phosphotransferase. The four newly described promoters and the previously established GAPDHp successfully initiated expression of the resistance gene and PPIp was selected for further marker development. The geneticin G418 resistance (aminoglycoside 3’-phosphotransferase, APH) and the nourseothricin resistance gene N-acetyl transferase (NAT) were tested for applicability in *C. oleaginosus*. Both markers showed high transformation efficiency, positive rate, and were compatible for combined use in a successive and simultaneous manner.

**Conclusions:**

The implementation of four endogenous promoters and one novel dominant resistance markers for *C. oleaginosus* opens up new opportunities for genetic engineering and strain development. In combination with recently developed methods for targeted genomic integration, the established toolbox allows a wide spectrum of new strategies for genetic and metabolic engineering of the industrially highly relevant yeast.

**Supplementary Information:**

The online version contains supplementary material available at 10.1186/s12896-023-00812-7.

## Background

*Cutaneotrichosporon oleaginosus* is an oleaginous yeast with the ability to metabolize a variety of both hexoses and pentoses and to produce high amounts of intracellular lipids. Among the known and described oleaginous yeasts it is one of the most efficient producers of single cell oils. Further, it has been shown to grow on a diverse range of industrial side streams like lignocellulosic hydrolysates [1, 2]. It can catabolize sugars without diauxic preferences and therefore generally shows a consistent growth behavior in the presence of several different carbon sources [3]. Further, in comparison to other yeasts, it is more resistant to toxic by-products of acidic hydrolysis, like furfural, HMF, organic acids, and phenols [4–6]. *C. oleaginosus* is capable of producing more than 80% lipid per dry cell weight and reaching lipid titers over 42 g/L in bioreactor fermentations [2, 7]. These qualities make it an ideal host organism for the conversion of side streams into valuable single cell oils and oleochemicals that can be used e.g., for biofuel, lubricant, cosmetic, and food applications.

Its unique properties make *C. oleaginosus* an attractive target for metabolic engineering to enhance triglyceride productivity, generate altered lipid profiles, change its ability for carbon substrate utilization, or implement lipid secretion [8–11]. Beyond triglyceride production, *C. oleaginosus* can be used as a cell factory platform to generate structurally diverse, high-value compounds [12]. *C. oleaginosus* is one of the most studied oleaginous yeasts, next to *Rhodotorula toruloides*, *Yarrowia lipolytica*, and *Lipomyces starkeyi* [9]. The single cell oils produced by these oleaginous microorganisms vary in terms of fatty acid composition, which qualifies them for different applications. *L. starkeyi* for instance, produces large amounts of palmitic acid (33.3%), followed by *R. toluroides* (20.0%). *R. toluroides* also shows a high amount of the essential fatty acid linoleic acid (13.1%), which is more than double the share found in single cell oil of the other three yeasts, and has added nutritional value [9]. Among those however, *C. oleaginosus* is one of the least studied with regard to the availability of genetic tools, which currently complicates its genetic optimization [8, 9, 13]. At present, no plasmid-based system is known for *C. oleaginosus*, which makes genetic modification dependent on time-consuming genomic integration methods. All these methods depend on reliable selection markers, whereof only a limited number is available for this yeast. Until recently, *Agrobacterium tumefaciens* mediated transformation (ATMT) and electroporation-mediated random integration techniques were the only vector-based methods to introduce DNA into the genome of *C. oleaginosus* [13, 14]. Transformation with *A. tumefaciens* is a successful and reliable method used for the introduction of genetic material into yeasts and fungi for almost three decades [15–17]. While ATMT is a fast transformation method, it does not allow targeted genomic integration. Specifically, *A. tumefaciens* randomly introduces multiple gene copies at different genomic locations. As the integration site cannot be controlled, genetic insertions can have detrimental effects on the growth of the transformants and on the expression of the gene(s) of interest. While ATMT was successfully used to generate non-native fatty acids in *C. oleaginosus*, we recently reported a CRISPR-Cas based approach for targeted genomic integration and deletion to yield mutants with modified fatty acid profiles [13, 18].

However, targeted genetic and metabolic engineering of microbial cell factories not only builds on reliable genetic accession tools but is also highly dependent on the availability of different antibiotic resistance cassettes, as well as functional promotor sequences to facilitate tunable, homologous, or heterologous gene expression. For *Yarrowia lipolytica* as well as *Rhodosporidum toruloides* native and engineered endogenous promoters as well as synthetic combinations have been characterized, which can be used for targeted metabolic engineering approaches [19, 20]. For metabolic engineering in *C. oleaginosus*, only a total of three endogenous promoters have been used for gene expression in previous studies [13, 18]. Of these, the glyceraldehyde 3-phosphate dehydrogenase promoter (GAPDHp) was used for the heterologous expression of a dominant marker and other genes of interest. The other two promoters used for homologous expression originated from the aldo-keto reductase (AKR) and the transcription elongation factor (TEF), which were combined with different endogenous genes of *C. oleaginosus* in our previous work [18]. In the same study, also three native genes (URA5, D9FAD and D12FAD) were used for genetic engineering [18]. As *C. oleaginosus* is commonly used for lipid accumulation, promoters should be especially active under these conditions to be used for metabolic engineering. However, strongly reduced expression behavior was reported for the GAPDH promoter under nitrogen limitation, which is commonly used to induce accumulation of lipids [13]. The available promoters currently not allow diversified genetic engineering approaches to build a platform for oleochemical production beyond tailored triglycerides [12]. Consequently, to allow for more flexibility in genetic engineering approaches, the availability of a larger selection of promoters for *C. oleaginosus* to expand the molecular biology toolbox would be highly desirable. To that end, specifically constitutive promoters which act independently of the respective metabolic situation would be an important addition to the toolbox of metabolic engineering of the oleaginous yeast.

To date, the low availability of promoters is not the only limiting factor for genetic engineering of *C. oleaginosus*. This issue extents to having only a few reliably working selection markers available including one auxotrophic marker (orotate phosphoribosyltransferase, URA5) for negative selection and three dominant resistance markers (HPH: hygromycin B phosphotransferase, PDR4: pleiotropic drug resistance 4, APH: aminoglycoside 3’-phosphotransferase) [13, 14, 18]. Auxotrophic markers allow for genetic engineering without antibiotics and mediate strong selection. However, a major drawback of using auxotrophic markers for selection is that an auxotrophic strain must be established before, which is often a rather work-intense effort. Also, once introduced, the markers should be expressed in the host at physiological levels, which can be difficult to balance [21]. To this end, dominant markers are a robust and faster alternative, because the wild type (wt) can be directly used for transformation. However, the introduction of large genes can result in a metabolic burden for the host cell, reduced growth, and productivity. Moreover, the antibiotics have to be well tolerated by the transformed cells [21].

In general, to introduce more than one genetic construct, it is useful to have several selection markers at hand. For other oleaginous yeasts like *Y. lipolytica* there are already several other auxotrophic markers such as *URA3*, *LEU2* and *LYS5* available, as well as dominant markers like the geneticin and nourseothricin resistance [22]. Both geneticin G418 and nourseothricin inhibit ribosomal translation [23, 24]. Geneticin G418 resistance can be mediated by the enzyme aminoglycoside 3 ‘phosphotransferase (APH). The transposon Tn903 from *Escherichia coli* carrying the APH sequence was established in *S. cerevisiae* as the resistance marker KanMX already in 1980 [23, 25]. In case of nourseothricin, the resistance gene for nourseothricin N-acetyl transferase (NAT) is found in the producer strain *Streptomyces noursei* and can be used as a dominant resistance marker [23, 26]. Both dominant markers have been successfully used and combined in genetic work using different conventional and oleaginous yeasts [22, 24].

The unconventional yeast *C. oleaginosus* is a promising candidate for the industrial production of single cell oils [9]. However, to allow efficient metabolic and genetic engineering strategies, it is required to extend the molecular biology toolbox for this microorganism. Until now, only a few promotors and selection markers have been used. The goal of this study was to extend the portfolio of the available genetic tools. Therefore, five endogenous promoters were tested for their activity for heterologous gene expression. Furthermore, the cytotoxic effect of the two antibiotics geneticin G418 and nourseothricin was investigated on *C. oleaginosus* wt cells. Finally, *A. tumefaciens*-mediated transformation was chosen as a convenient method to evaluate geneticin G418 and nourseothricin resistances as markers for positive selection in *C. oleaginosus* and to further assess their compatibility. The experiments aim at extending the current metabolic engineering tools available for *C. oleaginosus* to access the potential of this oleaginous yeast for the sustainable production of single cell oils and oleochemicals.

## Results and discussion

### In silico analysis of *Cutaneotrichosporon oleaginosus* promoter elements

To increase the number of available promoters for genetic engineering approaches, the corresponding regulatory elements had to be identified. However, gene regulation in eukaryotic organisms is still not fully understood, especially for non-model organisms like the oleaginous yeast *Cutaneotrichosporon oleaginosus*. Fortunately, modern sequencing technology simplifies access to genetic information. The genome of *C. oleaginosus* was sequenced and the corresponding transcriptome was annotated in 2015 by Kourist et al. [27]. These genomic and transcriptomic data were used for the *in-silico* analysis of promoter and regulatory elements of the non-conventional yeast. Known endogenous yeast promoters commonly consist of a core promoter, comprising the minimal sequences required for the transcription start, and further different regulatory elements, including the proximal promoter as well as regulatory upstream activation sequences (UAS) [19]. The core promoter in yeasts usually contains one or several transcription start sites (TSS) and a non-obligatory TATA-box upstream of the start codon [28–30].

### TATA-box screening

For TATA-box motif screening, a dataset of up to 310 genes was selected from the highest expressed genes in *C. oleaginosus* cultivated with glucose as carbon source, as obtained from proteomics analysis of Fuchs et al. in 2021 [5]. Several sequence areas relative to the mRNA start (-800 to -1, -200 to -1, and − 100 to -1) were selected with RSAT Fungi ‘retrieve sequence’ tool, mostly excluding other overlapping elements [31]. These sequence sets were analyzed with the MEME tool for motif discovery. Different settings for motif occurrence and motif lengths of 5 to 8 bp were tested [32]. Furthermore, other tools (FIMO and MAST) were employed to search for specific TATA-motifs known from *Saccharomyces cerevisiae* and other oleaginous yeasts [33]. However, no significant conserved TATA-box-like sequence could be identified. Therefore, the TATA-box annotation was neglected for the promoter description.

In this context, motif discovery with the MEME tool certainly has its limitations, specifically for short sequence motifs, such as the TATA-box, a strong conservation is required to find statistically significant results in a dataset. Further, searching for known motifs originating from other yeast employing tools like MAST, can only identify patterns relevant for the corresponding yeast species. However, the transferability of these motif search algorithms from known species to genetically poorly understood organisms, such as *C. oleaginosus* might be very limited. Consequently, an enrichment of A and T bases at specific positions relative to the TSS, might not be identified with this approach but could nevertheless exist.

### Search for transcription start site

The TSS can either be positioned before or around the start codon, and in the latter case, it is at the same position as the translation initiation site (TIS). However, the mRNA can also start with the first intron of a gene, the 5’-untranslated region (UTR) [28]. To this end, the importance of the intronic region for promoter activity has already been shown in the oleaginous yeast *Rhodotorula toruloides* [28]. The search for conserved TSS motifs in this study was performed with the data set of up to 310 highly expressed genes as mentioned above. The area from − 50 to + 50 relative to the annotated mRNA start was selected with RSAT [31]. However, the motif search with MEME did not reveal a statistically relevant sequence that exclusively occurs in the potential TSS region. Therefore, no TSS was annotated.

An obstacle that can influence the TSS motif search is the mRNA annotation. The mRNA is annotated from transcriptomics data and therefore has already been processed by the cell [34]. Hence, the exact position of the TSS for each gene is hard to identify, which makes the motif discovery relative to the mRNA start difficult. Surprisingly, during the search for the TATA-box and TSS motifs, both a general putative regulatory element and a motif for the TIS were found and further analyzed.

### Discovery of CT-rich motif

**Fig. 1:**
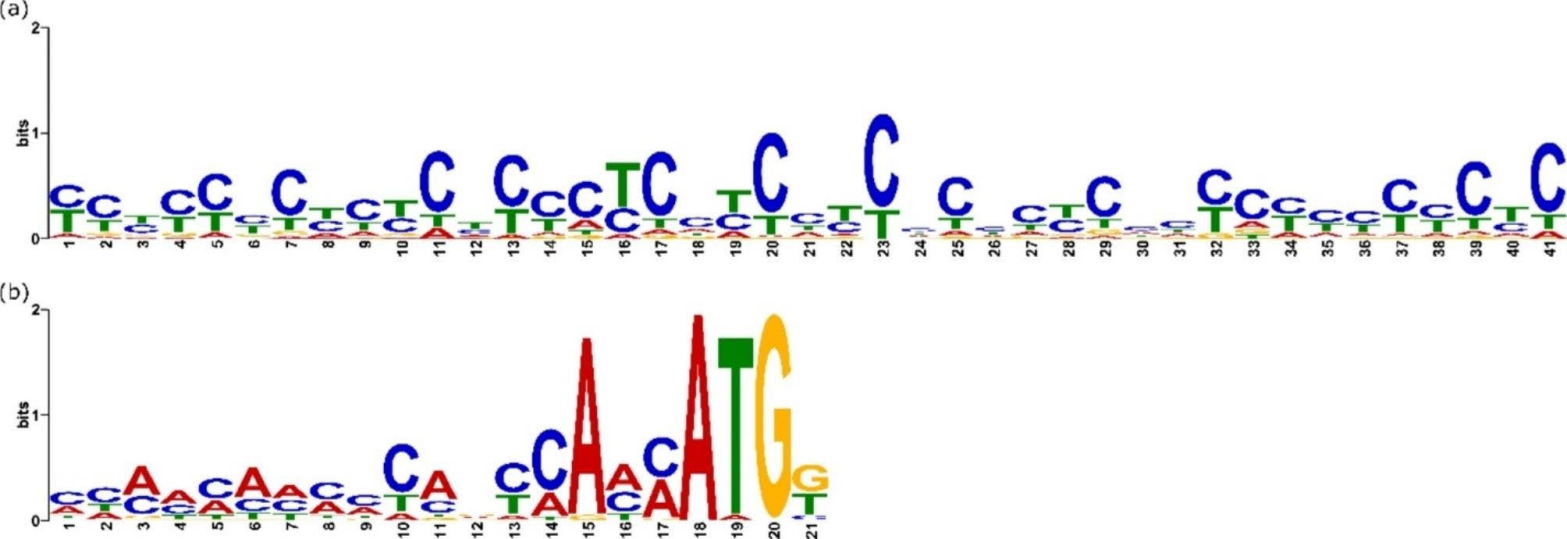
(**a**) 41 bp long CT-rich stretch, that was found in the promoter region in 245 sites within the 298 sequences of high expressed genes from *C. oleaginosus* with statistical significance with an E-value of 2.3e^− 227^. (**b**) The translational initiation site of 21 bp Kozak-like sequence was found in 54 of 301 sequences and is located from − 17 to + 4, relative to the start codon ATG, with an E-value of 2.0e^− 40^

Regulatory elements like the UAS can be versatile in position, length, orientation, and sequence [19]. Mostly, regulatory elements are recognition sites for transcription factors (TF), cofactors, RNA binding or act as (steric) their regulation sequences for [35–37]. The above-mentioned dataset of up to 310 genes was searched for these motifs. For this set the default RSAT settings were used to select the upstream sequence of the genes (-800 bp, excluding other annotated sequences, to -1). For 298 genes sequences longer than 8 bp were found and were included. Subsequently, a highly significant element could be identified, comprising a 41 bp long CT-rich motif, with an E-value of 2.3e^− 227^. It was identified in 245 sites within 189 of the 298 sequences, with a position p-value less than 0.0001. The consensus sequence of the motif is YYYYYCYCYCYCCCYCCYCHYCHCYCYCHCYCYYYYYCYC, with Y coding for CTA and H for ACT, compare Fig. 1a. Notably, the CT-motif is most abundant in areas close to the gene start in 5’ to 3’ direction but was also found further upstream of the mRNA start as well as in the reverse direction, as shown in Figure S2.

However, the sequences are not necessarily restricted to the length of 41 bp, as very similar motifs are found differing in length, using different settings in the same or different sequence sets. The minimal and maximal length of the CT-rich motif is rather hard to specify. CT-rich stretches of over 100 bp, representing the potential maximal length, were identified manually within the data sets. The minimal length is challenging to identify as well, because some statistically relevant shorter motifs were found, but were always observed to be part of longer CT-rich regions. Repeating patterns were found comprising 4 to 12 bp. Therefore, the element can be summarized as a CT-rich repeat, accumulating in the areas before transcriptional initiation. The function of the repeats can be hypothesized to be of regulatory nature, like TF binding. Furthermore, for other basidiomycota related CT-motifs and repeats in promoter regions were described before, like for *Ceriporiopsis subvermispora* and *Pleurotus ostreatus* [38]. Nevertheless, the molecular function of these is not yet unraveled. Therefore, other approaches, aiming at the screening of TF binding site should be applied for a better understanding of the regulatory elements of *C. oleaginosus* promoters, such as DNA microarrays or Chip-seq [36, 39].

### Translational initiation site identification

The translational initiation is coupled to the binding of ribosomes to the mRNA, therefore it does not only require a specific sequence but also a certain tertiary structure of the RNA [40]. In this study we only focused on the genetic sequence around the translational start site, specifically the ATG start codon. A sequence selection of -30 to + 10 relative to the coding sequences (CDS) from the 310 gene-set described above was selected with RSAT and used for the sequence identification of the TIS. A conserved 21 bp motif was found at 54 sites within 301 provided sequences, with an E-value of 2.0e^− 40^. The sequence spans from − 17 to + 4, relative to the start codon ATG. The motif consists of an A- and C-rich stretch from − 17 to -7, a non-conserved position − 5 and a highly conserved A at position − 3. Position − 2 and − 1 again are AC-rich and the conserved ATG is followed by a G or T in most cases. The consensus sequence is MYMMMAHMMCAVYCAMMATGK (with M: CA, Y: CAT, H: ACT, V: GAC, K:GT), shown in Fig. 1b.

The start codon ATG is a conserved sequence for gene regulation across the kingdoms, for translational initiation the small ribosomal subunit (40 S) scans the mRNA from the m7G cap until the start codon is reached [41]. This recognition is also influenced by the surrounding nucleotide sequence, the TIS [40]. The TIS motif found in *C. oleaginosus* is related to the general Kozak motif described for many eukaryotic organisms by Kozak (1989) [42]. In the Kozak motif also the nucleotide − 3 and + 4 are highly conserved with an optimal recognition of the start codon with the sequence GCCRCCaugG (R = A/G) [41]. The motif found in *C. oleaginosus* follows this structure with the exclusion of the − 6 G in the Kozak sequence and a stronger emphasis on A in the position − 3. The cropped sequence in a Kozak-like format is VYCAMMaugK (M: CA, Y: CAT, V: GAC, K:GT).

### Endogenous promoter for the heterologous gene expression in *Cutaneotrichosporon oleaginosus*

To identify putative endogenous promoters of *C. oleaginosus* for heterologous gene expression, suitable genes were manually selected according to the expression rates in the transcriptomics data published by Kourist et al. in 2015 [27]. As criteria transcription levels in both glucose media and in nitrogen-limited conditions were considered as well as the biological function of the genes. Based on these criteria, the five endogenous promoters GAPDHp, D9FADp, UBIp, PPIp, and 60Sp were selected for this study as listed in Table 1. The GAPDH promoter was the only one described in literature and used in a longer 800 bp version, as well as in a cropped 390 bp version [13]. For this study, the 800 bp version was employed.

The length of the novel putative promoters D9FAD, PPIp, and UBIp was selected to be the sequence upstream of the start codon at least including the annotated 5’UTR region. Further, the next CT-rich repeats region upstream of the 5’UTR was included in the promoter sequence. This resulted in respective promoter lengths of 191 bp for D9FADp, 154 bp for PPIp and 463 bp for UBIp. The 60 S promoter structure differed clearly from the others containing a less conserved region of CT-rich repeats, no 5’UTR and no ATG start codon at the beginning of the annotated CDS. It was, therefore, tentatively selected with 421 bp upstream of the annotated gene start. FIMO was employed for the annotation of the TIS, and CT-rich repeats described above [33]. The predicted TIS and the CT-motif identified before were used as indicators for the promoter annotation, as shown in Fig. 2a. In four of the five selected genes TIS motifs were identified according to the Kozak-like sequence. Only in the 60Sp gene no TIS motif was identified. The 41 bp long CT-motif was identified in all annotated sequences. In the promoter of GAPDH 96 hits for the CT-motif were found with a p-value < 10^− 4^, 76 hits in D9FADp, 70 hits in UBIp, 31 hits in PPIp and only 4 in 60Sp. The number and length of the CT-rich regions that span over all motif hits vary in each promoter sequence from 54 to 134, as listed in Table 1. The coverage of the regions with CT-motif hits is further different for each sequence, but quite high for GAPDHp, UBIp, D9FADp, and PPIp, but with only 4 hits on a 113 bp region very low for the 60Sp. For a clearer visualization only the CT-rich regions, including the identified CT-motifs, were annotated in Fig. 2b. As an example for the annotation of the CT-motif, Figure S1 shows the structure of the PPI promoter with all overlapping CT-motifs annotated by FIMO and the resulting CT-rich region stretches.

The annotations strengthen the hypothesis that the CT-motifs might be important for TF binding in the core promoter region in *C. oleaginosus*. In all promoters selected in this study, the motifs were cumulatively annotated in the region from 450 bp (mainly 200 bp) upstream, to the start of the CDS. In four of the five promoters a 5’UTR is annotated upstream of the start codon. In the case of UBIp there is a 5’ intron annotated included within the putative promoter region. All 5’UTR regions contain CT-motifs, this is in accordance with literature that showed that the 5’UTR and intronic regions are important for the promoter activity in other non-conventional yeast [28].

**Table 1:** Selection of promoters from endogenous genes of *C. oleaginosus*. UBIp, 60Sp, D9FADp and PPIp were newly identified in this study

#	Promoter	Gene of origin	Gene ID	Length selected	5’UTR	Count of CT-motifs (41 bp)	Length of CT-rich regions (bp)
1	GAPDHp	Glyceraldehyde 3-phosphate dehydrogenase	276,138	800	yes	96	134, 82
2	UBIp	Ubiquitin and ubiquitin-like proteins	289,369	463	yes	70	59, 96, 82, 66
3	60Sp	60 S ribosomal protein L37	224,393	421	no	4	113
4	D9FADp	delta-9 fatty acid desaturase	308,253	191	yes	76	66, 54
5	PPIp	Cyclophilin type peptidyl-prolyl cis-trans isomerase	286,945	154	yes	31	74, 51

**Fig. 2:**
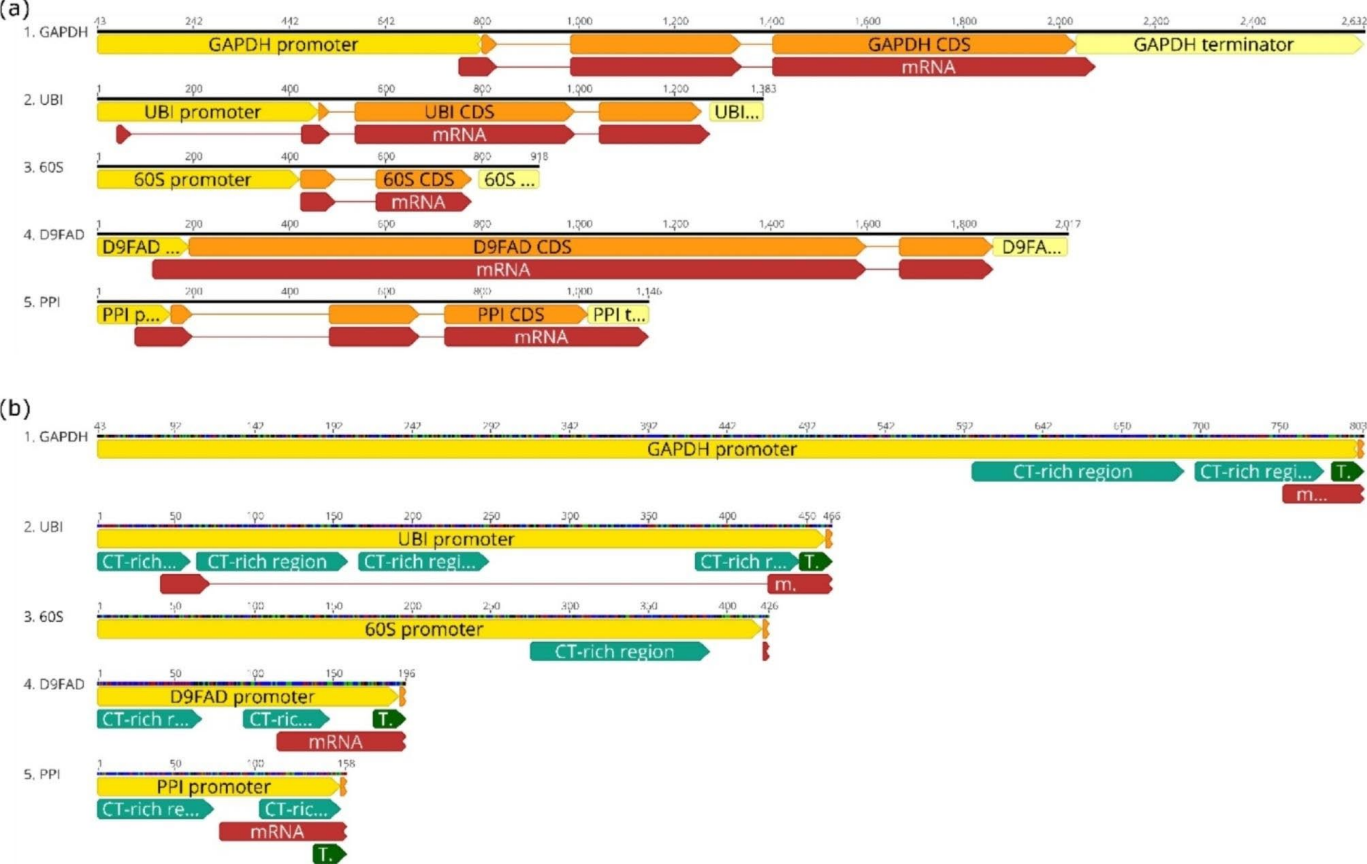
(**a**) Annotation of selected genes from *C. oleaginosus*, with promoters (yellow), terminators (light yellow), CDS (orange) and mRNA (red) annotated by Kourist et al. [3]. Putative promoters were annotated according to CT-motifs identified in this study and including the 5’UTR, shown in detail in (**b**). The CT-rich regions (green) within the putative promoter regions indicate where CT-motifs were identified. The identified TIS motifs (dark green) as well as the mRNA transcripts (red) and the first 4 bases of the CDS (orange) are also depicted

### Evaluation of different promoters for functional gene expression in *Cutaneotrichosporon oleaginosus*

**Fig. 3:**
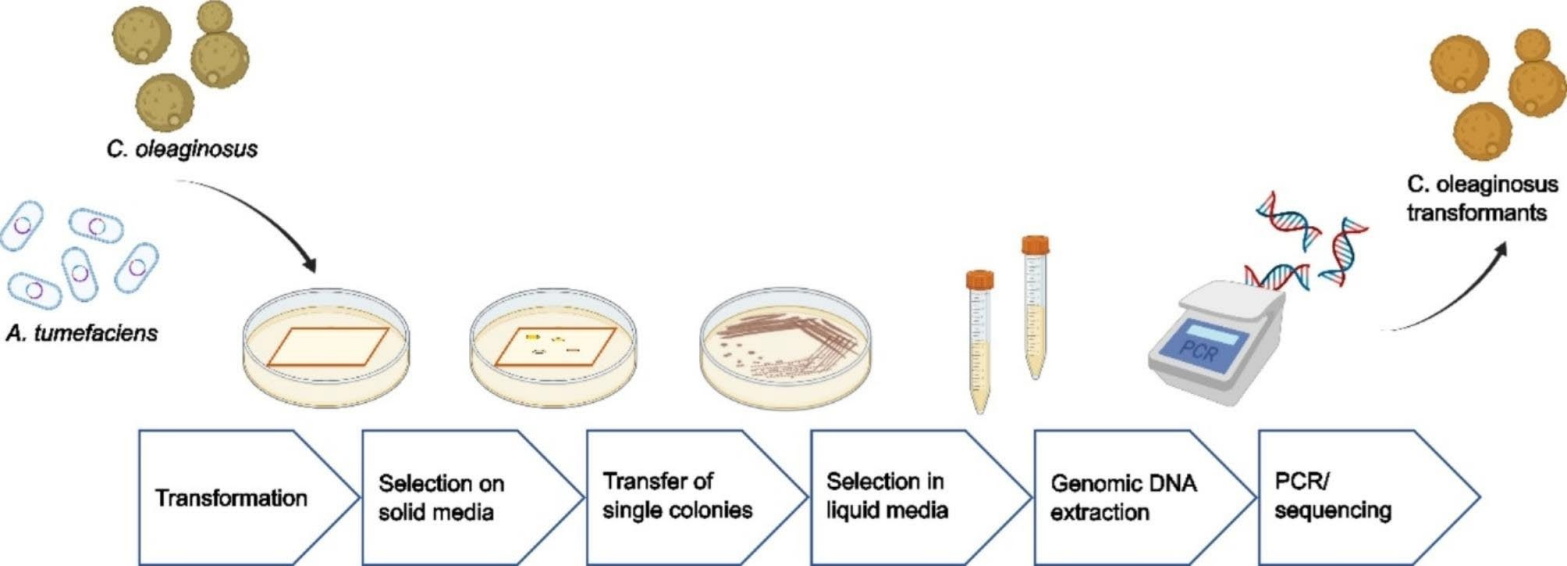
Workflow for the genetic modification of *C. oleaginosus* with an *Agrobacterium tumefaciens-*mediated transformation. *A. tumefaciens* cells with a plasmid containing a resistance were used to transfect *C. oleaginosus* followed by selection on solid media. After restreaking single colonies were used to inoculate liquid media. Finally, the genomic DNA was extracted for PCR amplification of the respective gene and sequencing for validation. Parts of the figure were created with BioRender

Of the five promoters selected as described above, only GAPDHp was previously described as a promoter for heterologous gene expression in *C. oleaginosus*. The promoter was applied in a modified pRF-HU2 plasmid for *Agrobacteria*-mediated transformation to produce non-native fatty acids in *C. oleaginosus*. The method is summarized in Fig. 3 [13]. This construct contained the hygromycin B phosphotransferase (HPH) gene from the pRF-HU2 plasmid, originating from the bacterium *Streptomyces hygroscopicus*. The HPH gene mediates resistance towards hygromycin B, an aminoglycoside, which inhibits protein biosynthesis.

**Fig. 4:**
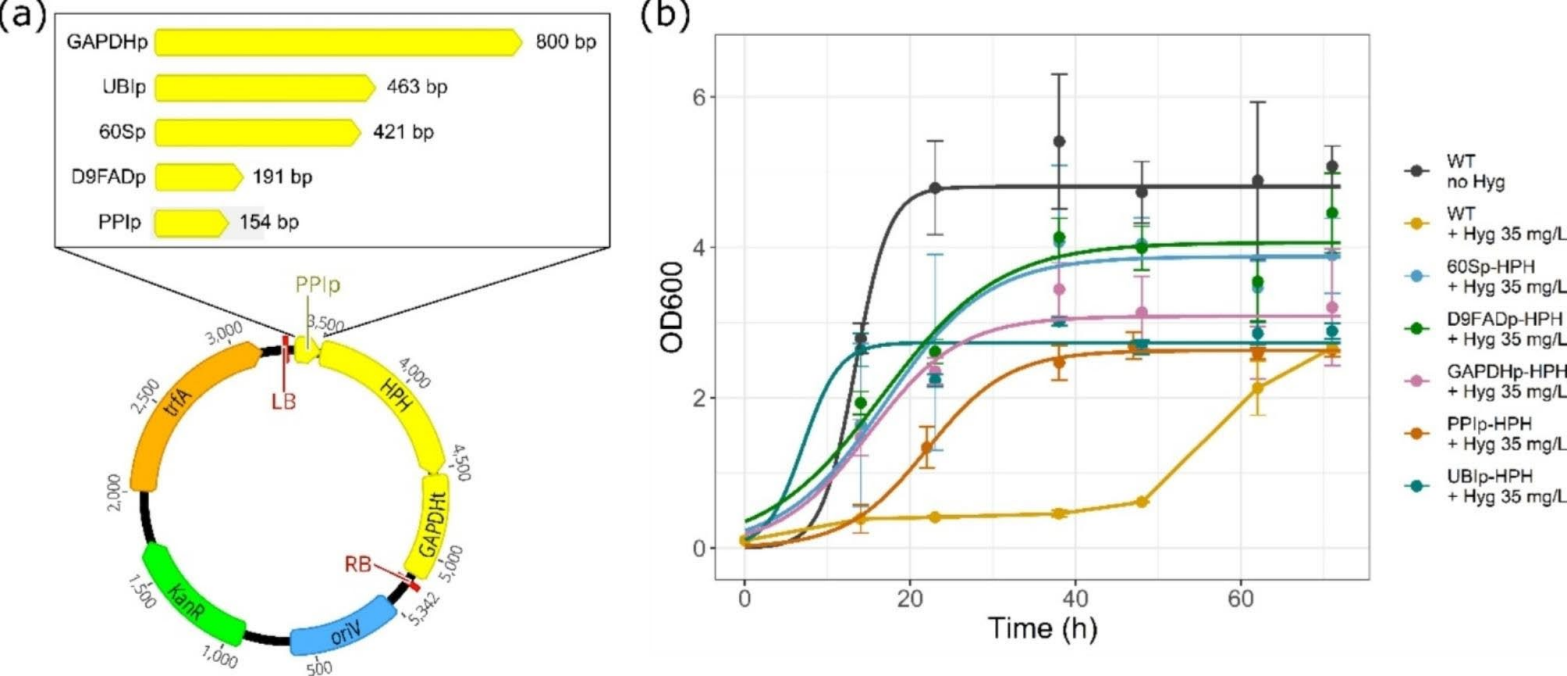
(**a**) pRF_Xp_HPH vector used for ATMT of *C. oleaginosus* with the five different promoters tested. Elements for plasmid replication and amplification in *E. coli* include KanR: kanamycin resistance; oriV: plasmid origin of replication; trfA: plasmid replication initiator protein. (**b**) Growth of *C. oleaginosus* mutants, with genomically integrated promoter-HPH constructs, and wt cells as controls with and without the addition of hygromycin B. The measurements were performed with four biological replicates. 60Sp, 60 S ribosomal protein L37 promoter; D9FADp, delta-9 fatty acid desaturase promoter; GAPDHp, glyceraldehyde 3-phosphate dehydrogenase promoter; HPH, hygromycin phosphotransferase; Hyg, hygromycin B; PPIp, cyclophilin type peptidyl-prolyl cis-trans isomerase promoter; UBIp, ubiquitin and ubiquitin-like protein promoter; wt, wild type. Plasmid maps were created with Geneious

To further assess which of the four putative promoters identified in this study would initiate gene expression, GAPDHp was replaced with D9FADp, UBIp, PPIp, and 60Sp, which were amplified from genomic DNA of *C. oleaginosus.* The GAPDH terminator was kept as a regulatory element for all constructs, resulting in the plasmids pRF-D9FADp-HPH, pRF-UBIp-HPH, pRF-PPIp-HPH, and pRF-60Sp-HPH, described in Fig. 4a. The constructs were all subsequently tested by PCR for, potentially genomic, integration via ATMT of *C. oleaginosus*. The transformation of *C. oleaginosus* wt cells with all plasmids resulted in CFUs on the membrane in the first round of selection. These CFUs were then transferred and streaked out on YPD plates. Single colonies from the plates were subsequently used to inoculate hygromycin B-containing YPD broth. All the tested plasmids resulted in transformants capable of surviving in the presence of the antibiotic in liquid media, reaching similar ODs as the wt without exposure to hygromycin B. Figure 4b shows the growth behavior of the mean of four biological replicates for each construct in comparison to the wt with and without the addition of hygromycin B. The wt without addition of hygromycin B outperformed the mutants in all cases. The growth of the wt yeast was suppressed in the presence of hygromycin B at 35 µg/mL only up to 46 h after inoculation.

This allows for the conclusion that *C. oleaginosus* shows some inherent resistance against the antibiotic, making it prone to lead to false positive results. The high variability in growth between the five promoters and the wt may result from different activities of the promoters. However, a high variability between the mutants using the same construct was observed, resulting in a high standard deviation. In ATMT, genomic integration takes place in an untargeted way, leading to high biological variability and difficulties with the quantification of the promoter activity. This might result from differences in the transcription frequency within the different loci of gene integration. In addition, the number of integrations can vary between the transformants, resulting in different gene copy numbers. Growth on solid and in liquid media was also not stable for some of the mutants and some could not be re-cultivated after storage. Overall, the observations characterize the ATMT using hygromycin B as potentially error-prone and inconsistent. Under these circumstances, evaluation of the quantitative promoter strength is difficult, which was the reason that methods such as quantitative PCR or the expression of fluorescent proteins were not applied. The results further show, that there is a strong need for alternative dominant markers for *C. oleaginosus*, that show a lower background and produce more stable transformants. This led to the characterization of other dominant markers for *C. oleaginosus* in the following steps with a focus on selectivity and reproducibility for genetic modification.

Of the four promoter sequences newly identified, all initiated the expression of the HPH resistance marker. This confirms that the sequences were functional promoters, comprising at least the core regulatory elements. Hence, these promoters can be applied in future genetic engineering approaches in *C. oleaginosus*. Therefore, the applied methodology for promoter annotation qualifies for the identification of functional endogenous promoters, featuring elements like the described CT-rich repeats. Compared to other reported working promoters for gene expression in *C. oleaginosus*, UBIp, 60Sp, D9FADp, and PPIp are much shorter with PPIp being the shortest element with 154 bp. Of the four newly reported elements the longest was 463 bp (UBIp), which is still shorter compared to GAPDHp and AKRp with 800 bp, and TEFp with 913 bp previously described in literature for tailoring the fatty acid profile [13, 18]. Due to its small size, PPIp was used for further ATMT experiments in this study. This is an advantage as transformation efficiency might decrease with increasing size of the vector DNA, thus generally favoring shorter promoter sequences [43]. The four newly described promoter sequences for gene expression in *C. oleaginosus* are a valuable and convenient alternative to the long promoters described in previous studies. Having a larger set of different promoters is a requirement for more complex genetic engineering using several constructs because different promoters do not compete for the same TF.

### Dominant markers for the selection of genetically modified *Cutaneotrichosporon oleaginosus*

**Fig. 5:**
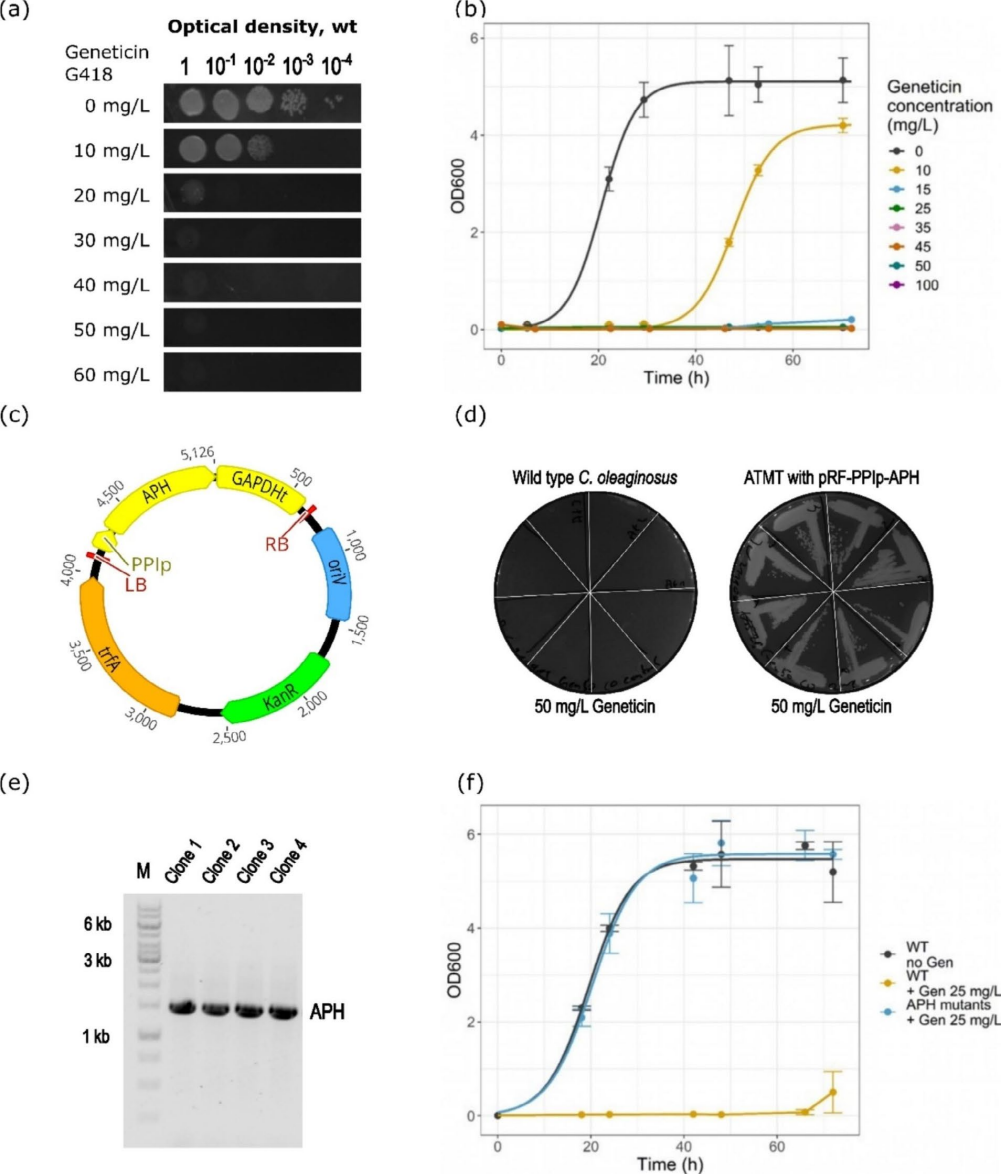
(**a**) Drop-test on solid media determining the minimal inhibitory concentration of geneticin G418 on *C. oleaginosus* wt. (**b**) Growth curves of *C. oleaginosus* wt with increasing concentrations of geneticin G418, determining the minimal inhibitory concentration in liquid media. (**c**) Map of the pRF_PPIp_APH plasmid, which was used for ATMT-derived integration of the APH resistance gene. Shown elements, besides the described gene cassette, include: KanR, bacterial kanamycin resistance; oriV, bacterial origin of replication; trfA, bacterial plasmid replication initiator protein. Plasmid maps were created with Geneious. (**d**) Restriped colonies after transformation of the wt control and the transformants on geneticin G418 plates. (**e**) Gel-electrophoresis of APH amplified by PCR (1599 bp) from genomic DNA of the transformants. The gel was cropped for clarity after lane 5, the complete gel picture is included in Figure S3. (**f**) Growth curves of the transformants and the wt, in triplicates, with and without the addition of geneticin. APH, aminoglycoside 3’-phosphotransferase; Gen, geneticin; wt, wild type

**Fig. 6:**
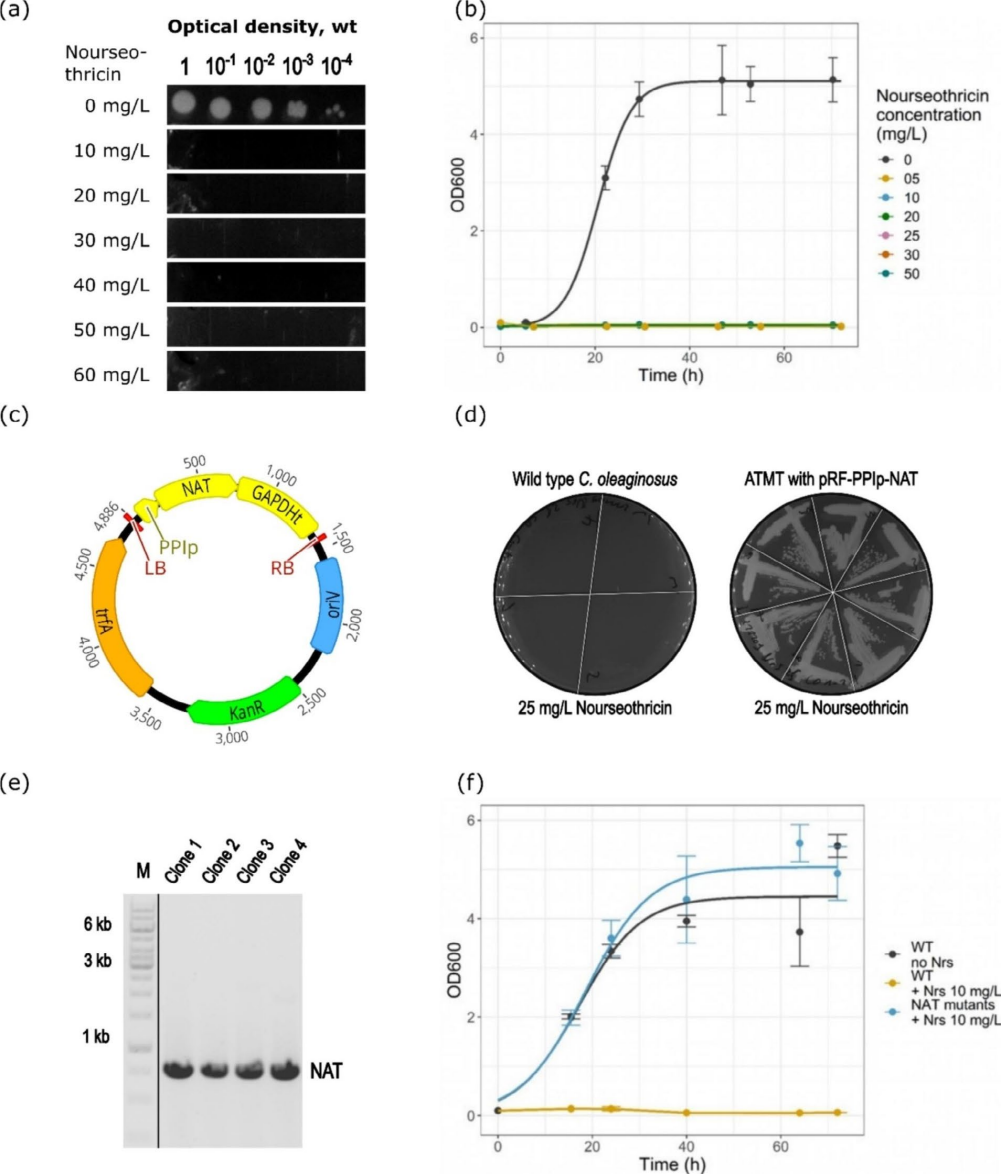
(**a**) Drop-test on solid media determining the minimal inhibitory concentration of nourseothricin on *C. oleaginosus* wt. (**b**) Growth curves of *C. oleaginosus* wt with increasing concentrations of nourseothricin, determining the minimal inhibitory concentration in liquid media. (**c**) Map of the pRF_PPIp_NAT plasmid, which was used for ATMT-derived integration of the NAT resistance gene. Shown elements, besides the described gene cassette, include: KanR, bacterial kanamycin resistance; oriV, bacterial origin of replication; trfA, bacterial plasmid replication initiator protein. Plasmid maps were created with Geneious. (d) Restriped colonies after transformation of the wt control and the transformants on nourseothricin plates. (**e**) Gel-electrophoresis of NAT amplified by PCR (783 bp) from genomic DNA of the transformants. The gel was cropped for clarity between the marker (**M**) and the lanes 2 to 5 and after lane 5, the complete gel picture is included in Figure S3. (**f**) Growth curves of the transformants and the wt, in triplicates, with and without the addition of nourseothricin. NAT, N-acetyl transferase; Nrs, nourseothricin; wt, wild type

The promoters GAPDHp, D9FADp, UBIp, PPIp, and 60Sp tested in this study all effectively initiated expression of the HPH protein. All further plasmids used for ATMT in this study featured the PPI promoter in front of the resistance marker. Until now, there was only one dominant marker described in the literature, the HPH resistance gene. This marker proved to have its difficulties, as false positive clones were a substantial part of the screening process and recultivation of the selected clones was sometimes not possible anymore after storage. Therefore, two dominant markers, known from genetic studies with other yeasts, were tested, namely APH and NAT. The CDS of the two enzymes was used to replace the HPH sequence in the plasmid pRF_PPIp-HPH. The sequence for APH originates from the bacterial transposon Tn903 and acts as geneticin G418 resistance marker. The NAT sequence comes from *Streptomyces noursei* and is a nourseothricin resistance marker. The sequences were retrieved from a public database, codon-optimized for expression in *C. oleaginosus*, and inserted into pRF_PPIp-HPH, resulting in the plasmids pRF_PPIp-APH and pRF_PPIp-NAT, as shown in Figs. 5c and 6c. To assess the applicability of these dominant resistance markers in *C. oleaginosus*, the cytotoxicity of geneticin G418 and nourseothricin against the unconventional yeast was tested on solid media, shown in Figs. 5a and 6a. To this end, a serial dilution of *C. oleaginosus* culture was dropped onto plates containing increasing concentrations of the respective antibiotic. The growth at 28 °C was monitored for two days. Based on the growth inhibition, a suitable concentration of the respective antibiotic was selected. Geneticin G418 completely inhibited growth at 50 mg/L and above, and nourseothricin at 20 mg/L and above. Therefore, for solid media selection 50 mg/L geneticin G418 and 25 mg/L nourseothricin were chosen. The growth behavior of *C. oleaginosus* wt was further assessed in YPD liquid media containing different concentrations of one of the antibiotics. In the presence of geneticin G418, growth of *C. oleaginosus* in YPD broth was fully inhibited at 25 mg/L, see Fig. 5b. In the case of nourseothricin, the growth was fully inhibited already at 10 mg/L, as displayed in Fig. 6b. The determined concentrations were used in all further growth experiments for liquid media selection using geneticin G418 or nourseothricin, respectively.

In the next step, ATMT was performed using pRF_PPIp-APH and pRF_PPIp-NAT. The concentration of antibiotics in the solid media for membrane transfer was doubled compared to the minimal inhibition concentration, following the common ATMT procedure described in Fig. 3. As substantial variation of the transformant numbers between the membranes of biological replicates is inherent to ATMT, the CFUs on the membranes were not analyzed quantitatively. From each of the three membranes 24 CFU were restreaked on solid media for single clone selection. *C. oleaginosus* wt transformed with the *A. tumefaciens* wt used as negative control did form up to 10 CFUs on the membranes in the presence of the respective antibiotic. The ATMT with pRF_PPIp-APH resulted in 65 out of 72 transformants growing on YPD plates with geneticin G418. In the case of pRF_PPIp-NAT, 70 out of 72 transformants survived on nourseothricin-containing plates. The streaked clones from the control ATMTs were not capable of surviving nourseothricin or geneticin selection on solid media (Figs. 5d and 6d). For the selection in liquid media, 32 transformants for each APH and NAT were used for inoculation of YPD with either nourseothricin or geneticin G418. For both dominant markers, all the selected CFU grew in liquid media in the presence of nourseothricin or geneticin G418, respectively (Figs. 5f and 6f). The integration was finally confirmed via PCR (Figs. 5e and 6e) and sequencing, leaving the uncertainty of the place of integration as, apart from a genomic site, an ectopic appearance of the DNA sequence might be possible. The results of the transformation experiments are summarized in Table 2.

The relatively high share of transformants (90% for APH and 97% for NAT) growing on plates with antibiotics allowed for an efficient transformation process with both dominant markers. The growth and validation of 100% of the *C. oleaginosus* mutants in liquid media showed that the approach leads to a reliable selection of transformants with confirmed, potentially genomic, integration of the marker. Furthermore, recovery from cryostocks worked consistently well. Thus, the experiments demonstrate that selection on solid media with the respective antibiotic is already a strong indicator for the integration of the genetic construct with APH or NAT. However, with 100% of the yeast clones surviving exposition to the antibiotic in liquid media, this allows for selection of transformants with a very low share of false positives, as confirmed by PCR. Overall, this study presents the use of the antibiotic resistance gene NAT for the genetic modification of *C. oleaginosus* for the first time. In comparison, both dominant markers qualify as reliable and efficient tools for selection using ATMT or any other transfection method for the genetic engineering of the unconventional yeast.

**Table 2:** Integration of the two dominant markers via ATMT into *C. oleaginosus*. Transformation efficiency, positive rate, and stability of the mutants are accessed by growth behavior and sequence confirmation. APH, aminoglycoside 3’-phosphotransferase; CFU, colony forming units; Gen, geneticin; NAT, N-acetyl transferase; Nrs, nourseothricin; suc, successive; sim, simultaneous

Dominant marker integrated into *C. oleaginosus*	Antibiotics added	Picked CFUs from three membranes	Growth on solid media + antibiotics	Growth in liquid media + antibiotics	Sequence confirmed
APH	Gen	72	65/72 (90%)	32/32 (100%)	3/3 (100%)
NAT	Nrs	72	70/72 (97%)	32/32 (100%)	3/3 (100%)
APH/NAT suc	Gen + Nrs	32	32/32 (100%)	18/18 (100%)	9/9 (100%)
NAT/APH suc	Gen + Nrs	32	30/32 (94%)	16/16 (100%)	4/4 (100%)
APH/NAT sim	Gen + Nrs	17	14/17 (82%)	11/11 (100%)	4/4 (100%)

### Combination of the two dominant markers

Until now, for the genetic modification of *C. oleaginosus*, only the HPH resistance gene has been established as a dominant maker. For the successive or simultaneous modification introducing more than one genetic construct, at least two compatible markers are required. APH and NAT are both aminoglycoside-modifying enzymes, but work with different molecule classes and have a different molecular reaction mechanism. APH is an aminoglycoside-3’-phosphotransferase phosphorylating an alcohol group of the target molecule geneticin G418. NAT is an N-acetyl transferase that acetylates the β-amino group of nourseothricin. Based on these differences, the two dominant markers could be compatible and might be used to independently integrate two separate constructs into the same target cell.

**Fig. 7:**
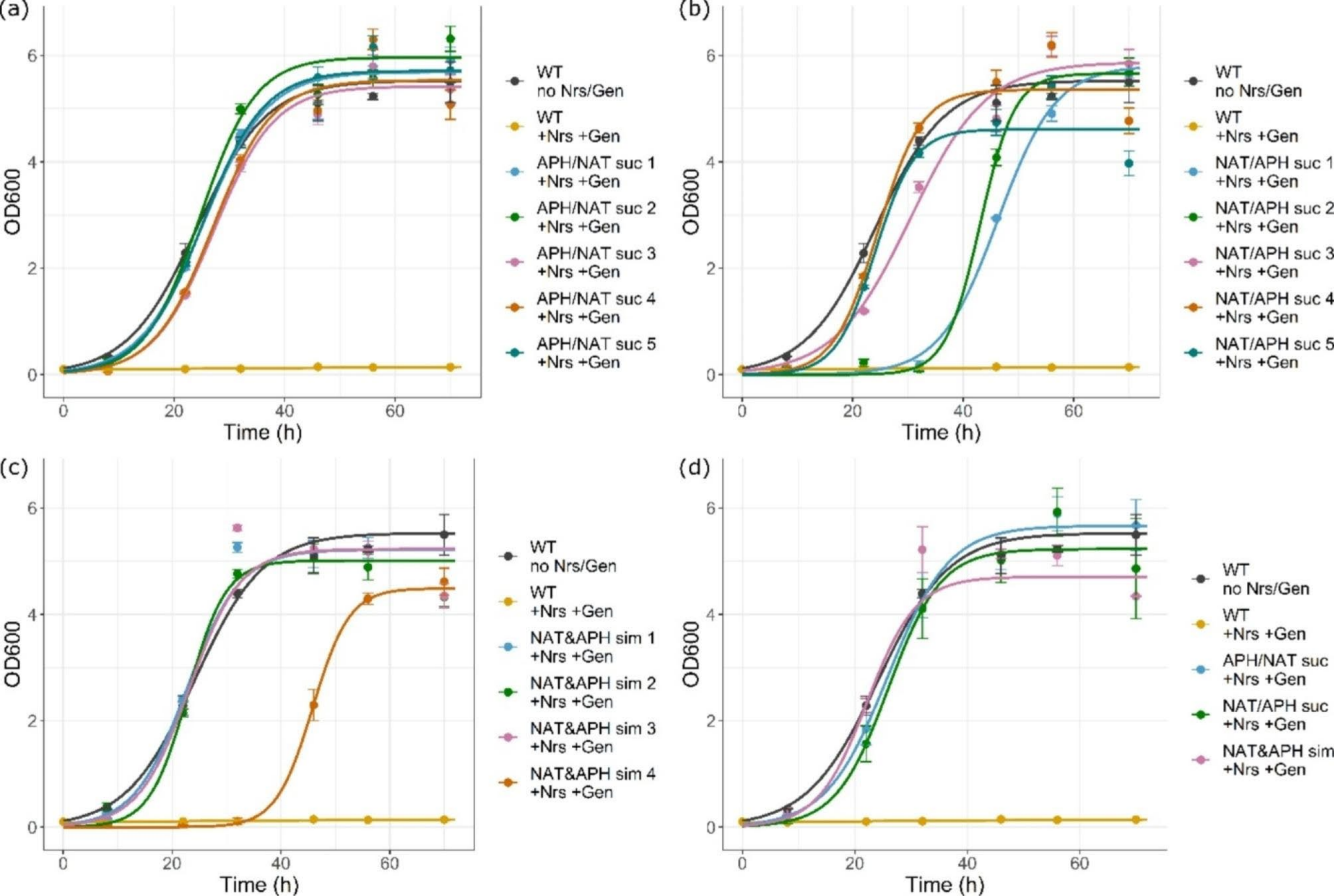
Growth curves of double mutants with the addition of both antibiotics (+ Nrs + Gen) in comparison to *C. oleaginosus* wt with and without the addition of both antibiotics. (**a**) Five double mutants from successive integration of APH/NAT in comparison to *C. oleaginosus* wt. (**b**) Five double mutants from successive integration of NAT/APH in comparison to *C. oleaginosus* wt. (**c**) Four double mutants from simultaneous integration of APH/NAT in comparison to *C. oleaginosus* wt. (**d**) Average from the biological replicates from (**a**, **b**, **c**), excluding the outliers with delayed growth behavior. APH, aminoglycoside 3’-phosphotransferase; Gen, geneticin; NAT, N-acetyl transferase; Nrs, nourseothricin; suc, successive; sim, simultaneous; wt, wild type

To assess this, APH and NAT were first tested for cross-reactivity. Transformants from ATMTs with pRF_PPIp-APH or pRF_PPIp-NAT were tested for cross-activity against nourseothricin or geneticin G418, respectively. No growth was observed on plates or in liquid media for transformants featuring either an integrated APH or NAT in presence of the respective other antibiotic. In a next step, the combination of the two marker genes was tested either with successive or simultaneous transformation. For successive transformation, a transformant with a confirmed genomically integrated APH or NAT was selected. The mutant was then used for an ATMT with the respective other dominant resistance marker. The efficiency of the cloning procedure is shown in Table 2. The transformation efficiency was high in the cases of successive transformation, CFUs on the membrane were more than 500 each for all three replicates. For the simultaneous transformation the efficiency was reduced significantly, with four to eight CFU on each transformation membrane. From the selected clones of the successive transformation of APH integration followed by NAT integration, all 32 grew (100%) on solid media containing both antibiotics. 18 further selected transformants were able to grow in liquid media. Further, all nine transformants used for genomic DNA extraction were positive for the gene sequence as shown by PCR and sequencing. The other successive combination, NAT followed by APH integration, was similarly successful. After restreaking, 30 out of 32 clones grew (94%) on solid media containing geneticin G418 and nourseothricin. All the 16 selected transformants survived in liquid broth with the antibiotics. The genomic DNA extracted from four of these clones featured both markers, as confirmed by PCR and sequencing. In case of the simultaneous integration of the APH and NAT marker, a total of 17 CFU on the three different transformation membranes were picked. Out of those 17 only 14 grew (82%) when restreaked on solid media, but from those all eleven, which were further selected, survived in liquid media. All four clones selected for sequencing tested positive for both markers. Liquid cultures for growth curves were measured for four to five of the verified mutants. *C. oleaginosus* wt in YPD without the addition of antibiotics was used as a reference, and a negative control with the wt yeast and the addition of 25 mg/L geneticin and 10 mg/L nourseothricin was cultivated as well. The mutants for the successive APH/NAT integration showed very stable growth in between the different mutants and were comparable to the wt in YPD without the antibiotics, as shown in Fig. 7a. Using the other successive order NAT/APH for combined integration the growth curves were less uniform. Two mutants showed a much longer lag-phase in growth, compared to the positive control, see Fig. 7b. For the simultaneous integration of both APH and NAT, three of the four mutants showed a growth behavior very similar to the positive control. One clone had a much longer lag-phase, and the fifth clone did not grow in the pre-culture and was therefore excluded. Figure 7c shows the growth behavior of *C. oleaginosus* transformants from a simultaneous integration of APH and NAT. The variability in between the biological replicates was higher, as one of the putative positive clones did not grow in recultivation conditions for the preculture, and therefore only four replicates were analyzed. All three transformation strategies were summarized for better comparison without the outliers in Fig. 7d.

The combinatory ATMT confirmed that APH and NAT show no cross-activity and thus can be considered compatible. The antibiotics could still be used to apply selection pressure on transformants which already had the respective other resistance gene integrated into their genome. This is in line with the findings in the literature for the yeast *Schizosaccharomyces pombe* [24]. Further, the successful integration of both markers in a successive as well as a simultaneous manner is possible and reliable. The method of selection and further cultivation of positive mutants is suitable for the generation of genetically modified *C. oleaginosus* using more than one construct, resulting in high rates of positives after primary selection of putative mutants from transformation. The order of the successive genetic modification is not crucial, but as the APH seems to show more variation, it is recommended to use the order APH/NAT. This way the variability between the generated mutants is lower than with the other order of markers. The transformational efficiency of the simultaneous integrated APH/NAT mutants was much lower, as the transfection and integration of two constructs at the same time is less likely than for one construct at a time. However, it can be a time-saving alternative to two separate ATMTs of *C. oleaginosus*.

## Conclusion

The non-conventional yeast *C. oleaginosus* has great potential as production organism for the biotechnological production of lipids and oleochemicals. Until recently, limited accounts on the genetic engineering of this yeast were published in the literature. However, metabolic engineering of *C. oleaginosus* might be of high value for the optimization of substrate adaptation, product specification, as well as growth optimization, and by-product variation. The genetic toolbox described in the literature was limited to three promoters with rather long sequences of up to 913 bp and one dominant resistance marker, which produced false positive transformants, and often could not be recultivated after storage. In this study, the sequences of putative endogenous promoters were analyzed based on more than 280 highly expressed genes of *C. oleaginosus*. The promoter regions of the genes featured a CT-rich motif which is suspected to have regulatory function in the oleaginous yeast. Based on this CT-rich motif and a TIS the four putative promoter sequences D9FADp, UBIp, PPIp, and 60Sp were selected and used for a qualitative expression tests in comparison to the known endogenous promoter GAPDHp. All putative promoters initiated the expression of the resistance gene HPH. PPIp (154 bp) as the shortest element was chosen for the construction of plasmids for ATMT with the two dominant markers APH and NAT. Both markers showed high positive rates (90% and 97% of the screened transformants, respectively) and resulted in stable mutants, which could be recultivated in the presence of geneticin G418 (in case of APH) or nourseothricin (in case of NAT). Selection by streaking and cultivation in liquid media resulted in transformants that were positive for the integration, which was finally confirmed via sequencing. This qualifies APH and NAT as functional dominant markers for the genetic modification of the non-conventional yeast. Furthermore, the compatibility of the two dominant markers was demonstrated. The two antibiotic-resistance genes were successfully integrated successively and simultaneously. In both combination orders, the successive integration had a high transformation efficiency, positive rate, and resulted in mutants with resistance to both markers. Transformation with both constructs at the same time in a simultaneous approach resulted in a reduced transformation efficiency, but both resistance genes were successfully integrated. Using ATMT as a screening method, the genetic toolbox of *C. oleaginosus* was considerably expanded with the novel endogenous promoters and dominant resistance markers. In combination with new targeted genomic integration methods as recently reported, the established tools from this study open up new opportunities for genetic and metabolic engineering of the industrially highly relevant yeast *C. oleaginosus*.

## Materials and methods

### Motif discovery

Genomic DNA sequences and transcriptional data of *C. oleaginosus* were used as published by Kourist et al. (2015) [3]. The selection of the 310 highest expressed genes was based on the proteomics data from Fuchs et al. (2021) with glucose as carbon source [5]. The extraction of specific sequence regions was performed with the RSAT retrieve sequence tool for fungi [31]. The regions were selected either relative to the start of the annotated coding sequence (CDS) or the mRNA start. TATA-box identification was mainly attempted with sequences from − 200 bp to -1 bp relative to the mRNA start. For TSS identification the region from − 30 bp to + 10 bp relative to the mRNA start was selected. UAS identification was done with the RSAT default settings, selecting − 800 bp to -1 bp (to mRNA) dismissing regions where other genetic elements are annotated. TIS annotation was performed with a dataset with 50 bp upstream and 50 bp downstream relative to CDS start. For motif discovery, the MEME suite was employed, with the MEME tool for novel motif discovery and FIMO for motif annotation within specific sequences [32, 33]. For motif discovery with MEME, default settings were used, with edited minimal motif length for the TATA-box search. FIMO was employed with default settings.

### Strain and cultivation on plates

The oleaginous yeast *Cutaneotrichosporon oleaginosus* (ATCC 20509 / DSM-11815) was retrieved from the Deutsche Sammlung von Mikroorganismen und Zellkultur (DMSZ, Braunschweig, Germany). *C. oleaginosus* was cultivated on agar plates to obtain single colonies for cultivation, mutant screening, recovery from cryo-stocks, and the determination of the minimal inhibition concentration of antibiotics. Yeast extract peptone dextrose (YPD) with 1.2% agar was used as solid medium for the plates, optionally with the addition of different concentrations of antibiotics. The plates were incubated at 28 °C for at least one day or up to three days until visible colonies appeared. For cell separation, screening, and recovery, cells were streaked out to obtain single colonies.

### Drop tests on plates

To assess the cytotoxicity and the minimal inhibitory concentration of different antibiotics towards *C. oleaginosus*, YPD plates were prepared with a dilution series of the respective antibiotics. YPD plates included 0–70 mg/L Geneticin sulfate G418 (Burlington, United States) or 0–60 mg/L nourseothricin (Carl Roth, Lausanne, Switzerland). An overnight *C. oleaginosus* culture in YPD was adjusted with fresh YPD to an OD_600_ of 1. From that, a serial dilution was prepared in steps with a factor of 10 until the minimal count of colony-forming units (CFU) on a YPD plate was around between 0.1 and 1 CFU/µL. 2 µL of each dilution was dropped on the antibiotic-containing plate. The plates were incubated at 28 °C for two days and the growth was documented with the CHEMI Premium Imager (VWR International, Germany).

### Cultivation in liquid media

Cultivation was performed in YPD broth and incubated at 28 °C while shaking at 120 rpm. When using shaking flasks 20% of the total volume was used for cultivation. Pre-cultures were inoculated with single colonies from agar plates, main cultures were inoculated to an OD_600_ of 0.1 from the pre-culture, measured with an Implen NanoPhotometer® (Implen, Munich, Germany). For growth curves, 24-well deep well plates were used with 2.5 mL culture in each well (total well volume of 10 mL) and incubated under humid conditions at 180 rpm shaking speed. OD_600_ was measured in 100 µL volume with the EnSpire™ Multimode Plate Reader by PerkinElmer Inc. in 96-well clear microtiter plates, diluted to a measured OD_600_ between 0.1 and 0.6.

### Promoters, resistance genes, and plasmids

To retrieve the promoter sequences for GAPDHp, 60Sp, PPIp, UBIp, and D9FADp primers flanking the identified promoter regions were used for amplification from genomic DNA, constructs are listed in Table 3. The primer sequences as well as the gene sequences are listed in the supplementary material Sequences.xslx. The plasmids featuring the HPH resistance gene (pRF_GAPDHp-HPH) were constructed based on a modified version of the plasmid pRF-HU2 previously described for *A. tumefaciens*-mediated transformation in *C. oleaginosus* [13]. The shuttle vector included the landing pads required for *A. tumefaciens*-mediated genomic integration as well as the bacterial kanamycin resistance cassette KanMX and the bacterial oriV system. GADPHp in the pRF_GAPDHp-HPH was replaced by any of the other promoters reported in this study, as indicated in the plasmid Table 3. The hygromycin B resistance gene in pRF_PPIp-HPH was replaced by the sequence of either APH or NAT, resulting in the two vectors pRF_PPIp-APH and pRF_PPIp-NAT. The amino acid sequence for APH as the geneticine G418 resistance was obtained from the established dominant kanamycin resistance marker transposon Tn903 (Uniprot ID: P00551). In the case of NAT as the nourseothricin resistance marker, the protein sequence of the enzyme from *Streptomyces noursei* was used (Uniprot ID: Q08414). The enzyme amino acid sequences were reversely translated using the Kazusa webtool (www.kazusa.or.jp/codon, 2018, Kazusa DNA Research Institute, Japan) for codon-optimized expression in *C. oleaginosus* and synthesized by Eurofins Genomics Germany GmbH (Ebersberg, Germany). Cloning of the plasmids was done by restriction cloning and/or Gibson assembly. The *Escherichia coli* laboratory strain DH5 α strain was used for selection and amplification of all constructed plasmids. The sequences were verified by Sanger sequencing by Eurofins Genomics Germany GmbH (Ebersberg, Germany).

**Table 3:** Plasmids used in this study with two dominant resistance makers under the control of different endogenous*C. oleaginosus* promoters

Plasmid no.	Plasmid name	Promoter regulating the resistance gene	Resistance gene	Origen
1	pRF_GAPDHp-HPH	Glyceraldehyde 3-phosphate dehydrogenase (GAPDH)	Hygromycin phosphotransferase (HPH)	Görner et al. [13]
2	pRF_D9FADp-HPH	Delta-9 Fatty acid desaturase (D9FAD)	Hygromycin phosphotransferase (HPH)	this study
3	pRF_UBIp-HPH	Ubiquitin and ubiquitin-like proteins (UBI)	Hygromycin phosphotransferase (HPH)	this study
4	pRF_PPIp-HPH	Cyclophilin type peptidyl-prolyl cis-trans isomerase (PPI)	Hygromycin phosphotransferase (HPH)	this study
5	pRF_60Sp-HPH	60 S ribosomal protein L37 (60 S)	Hygromycin phosphotransferase (HPH)	this study
6	pRF_PPIp-APH	Cyclophilin type peptidyl-prolyl cis-trans isomerase (PPI)	Aminoglycoside 3’-phosphotransferase (APH)	this study
7	pRF_PPIp-NAT	Cyclophilin type peptidyl-prolyl cis-trans isomerase (PPI)	Nourseothricin N-acetyl transferase (NAT)	this study

### *Agrobacterium tumefaciens*-mediated transformation

The integration of DNA sequences, potentially, into the genome of *C. oleaginosus* transformation was done with *Agrobacterium tumefaciens* according to Görner et al., 2016 [13]. In short, cells of *Agrobacterium tumefaciens* were transformed with the shuttle vector pRF containing the respective marker gene, using electroporation and selection on LB plates containing 30 µg/mL kanamycin. Overnight liquid cultures were then prepared from single colonies of the *A. tumefaciens* transformants in 5 mL LB medium with 30 µg/mL kanamycin at 28 °C. These cultures were used to inoculate 10 mL L-IMAS medium in shaking flasks, which were then incubated at 28 °C for 6 h. An overnight culture of *C. oleaginosus* was diluted to an OD_600_ of 0.5. 50 µl of the *C. oleaginosus* dilution and 50 µL of the 6-hour *A. tumefaciens* culture were mixed. The mixture was then plated onto an Amersham Hybond-N^+^ blotting membrane from GE Healthcare (Little Chalfont, United Kingdom) on top of an S-IMAS agar plate. The plates were incubated at 24 °C for 48 h and the membranes were subsequently transferred onto YPD agar plates containing an elevated concentration of the respective antibiotics: 150 µg/mL Hygromycin B (AppliChem GmbH, Darmstadt, Germany), 100 µg/mL geneticin G418 (Carl Roth GmbH, Karlsruhe, Germany) or 50 µg/mL nourseothricin (Carl Roth GmbH, Karlsruhe, Germany) or combinations thereof. To inhibit agrobacteria growth, also 300 µg/mL cefotaxime (Thermo Fisher Scientific Inc., Waltham, USA) was included in the plates. The method is visualized in Fig. 3.

### Screening of mutants

After transformation, single colonies were picked from the membrane and streaked out on YPD plates containing 300 µg/mL cefotaxime. To select for the respective antibiotic resistance 70 µg/mL hygromycin B, 50 µg/mL geneticin, or 25 µg/mL nourseothricin or combinations thereof were included in the solid media. Plates were incubated for two to three days until visible single colonies formed. Single colonies were then used for inoculation of 5 mL YPD medium supplemented with 35 µg/mL hygromycin B, 25 µg/mL geneticin G418, 10 µg/mL nourseothricin, or a combination thereof. Transformants that were able to grow in liquid cultures were pelleted and the genomic DNA was extracted using the Yeast DNA Extraction Kit from Thermo Fisher Scientific™ (Waltham, USA). PCR was then performed for the amplification of the DNA of the resistance markers and the results were versified via gel electrophoresis. Phusion™ High-Fidelity DNA Polymerase was employed and all reactions were performed with GC buffer and at annealing temperatures according to primer melting temperature. Elongation time was calculated with 20 s/kb for each individual fragment. Final verification of genomic gene integration was done by sanger sequencing at Eurofins Genomics GmbH (Ebersberg, Germany) after DNA purification with NEB Monarch® DNA Gel Extraction Kit (Ipswich, USA).

### Electronic supplementary material

Below is the link to the electronic supplementary material.

Supplementary Material 1

Supplementary Material 2

## Data Availability

The cloning vectors constructed during the current study are available in the GenBank repository, BankIt2716325, with the accession numbers: OR166493 (1_pRF_GAPDHp_HPH), OR166494 (2_pRF_D9FADp_HPH), OR166495 (3_pRF_UBIp_HPH), OR166496 (4_pRF_PPIp_HPH), OR166497 (5_pRF_60Sp_HPH), OR166498 (6_pRF_PPIp_APH), OR166499 (7_pRF_PPIp_NAT).

## Abbreviations

60Sp: 60 S ribosomal protein L37 promoter
AKR: aldo-keto reductase AKR
APH: aminoglycoside 3’-phosphotransferase
ATMT: *Agrobacterium tumefaciens*-mediated transformation
CFU: colony forming units
D9FADp: delta-9 fatty acid desaturase promoter
GAPDHp: glycerinaldehyde 3-phosphate dehydrogenase promoter
Gen: geneticin G418
HPH: hygromycin B phosphotransferase
KanR: kanamycin resistance cassette
NAT: N-acetyl transferase
Nrs: nourseothricin
PPIp: cyclophilin type peptidyl-prolyl cis-trans isomerase promoter
sim: simultaneous
suc: successive
TEF: transcription elongation factor
TF: transcription factor
TIS: translation initiation site
TSS: transcription start site
UAS: upstream activation sequences
UBIp: ubiquitin, and ubiquitin-like proteins promoter
UTR: untranslated region
wt: wild type
