# Supplementary material for: Expanding the genetic toolbox for Cutaneotrichosporon oleaginosus employing newly identified promoters and a novel antibiotic resistance marker
Source: BMC Biotechnol. 2023 Sep 18;23:40. doi: 10.1186/s12896-023-00812-7 (PMC10506223; doi:10.1186/s12896-023-00812-7)
Supplement: Supplementary file 2 — Supplementary Material 2 [file 12896_2023_812_MOESM2_ESM.pdf]

Supplementary material for

Expanding the genetic toolbox for *Cutaneotrichosporon oleaginosus* employing newly identified promoters and a novel antibiotic resistance marker

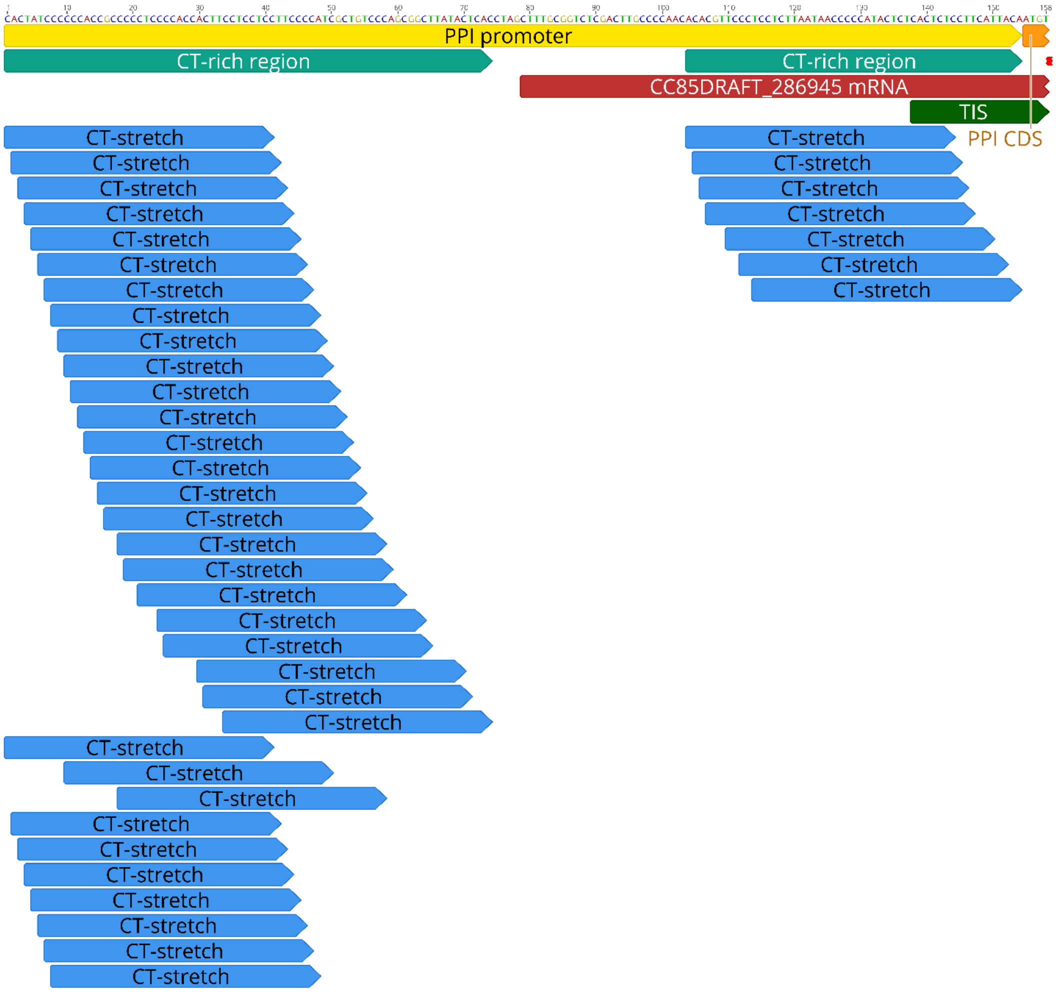

Figure S1. PPI promoter with all CT-motifs (CT-stretch, blue) identified by FIMO, as well as the resulting CT-rich region, comprising all hit regions (turquoise). mRNA is annotated (red), TIS (green), and the CDS (orange). CDS, coding sequence; PPI, Cyclophilin type peptidyl-prolyl cis-trans isomerase; TIS, translation initiation site.

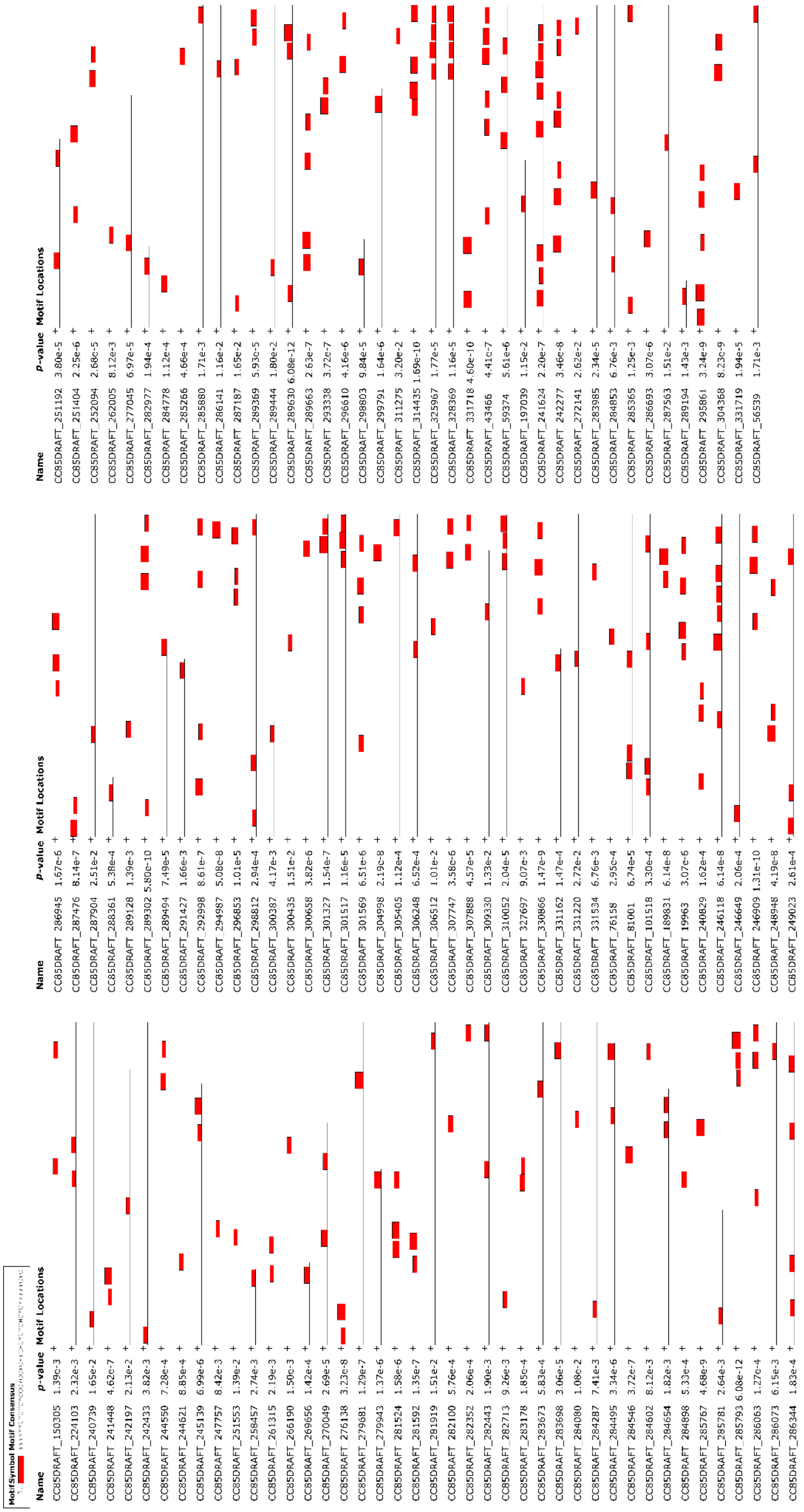

Figure S2. Localization of the 41 bp CT-motif in 189 hits of the 298-sequence data set with highly expressed *C. oleaginosus* genes.

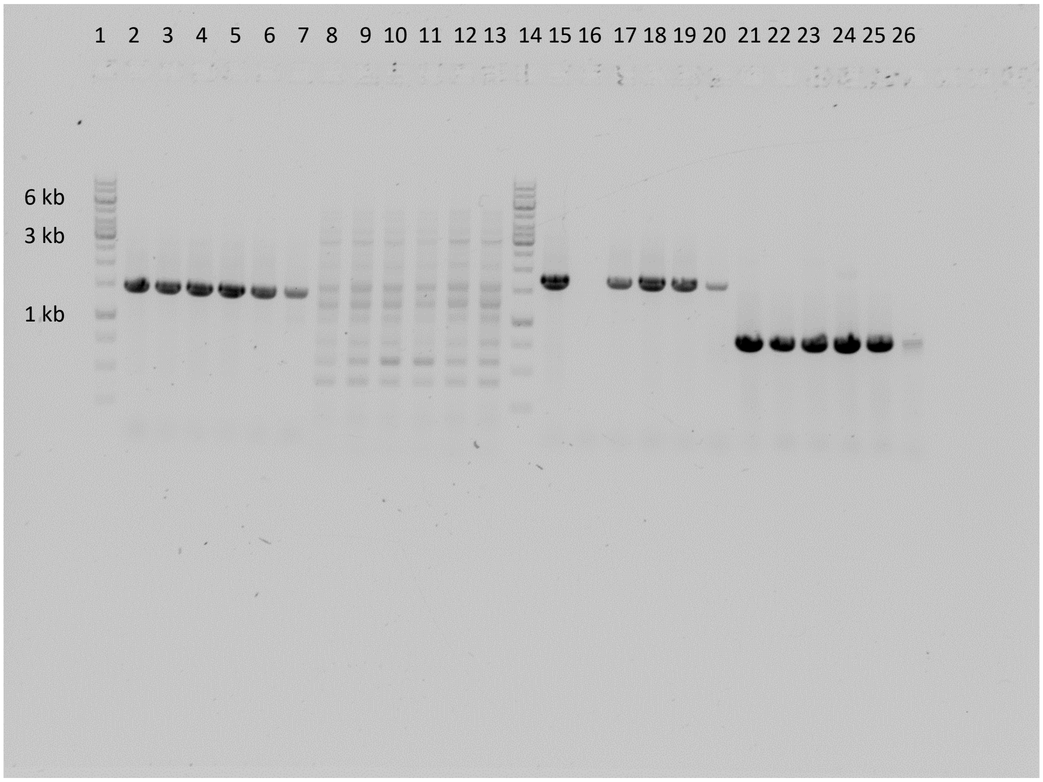

Figure S3. Uncropped gel-electrophoresis picture with the validation of the genomic integration of both APH and NAT gene into the *C. oleaginosus* genome. Lane 1: GeneRuler 1kb (ThermoFischer); lane 2-7: amplicon from genomically integrated APH gene; lane 8-13: unrelated; lane 14: GeneRuler 1kb (ThermoFischer); lane 15-20: unrelated; lane 21-26: amplicon from genomically integrated NAT gene.

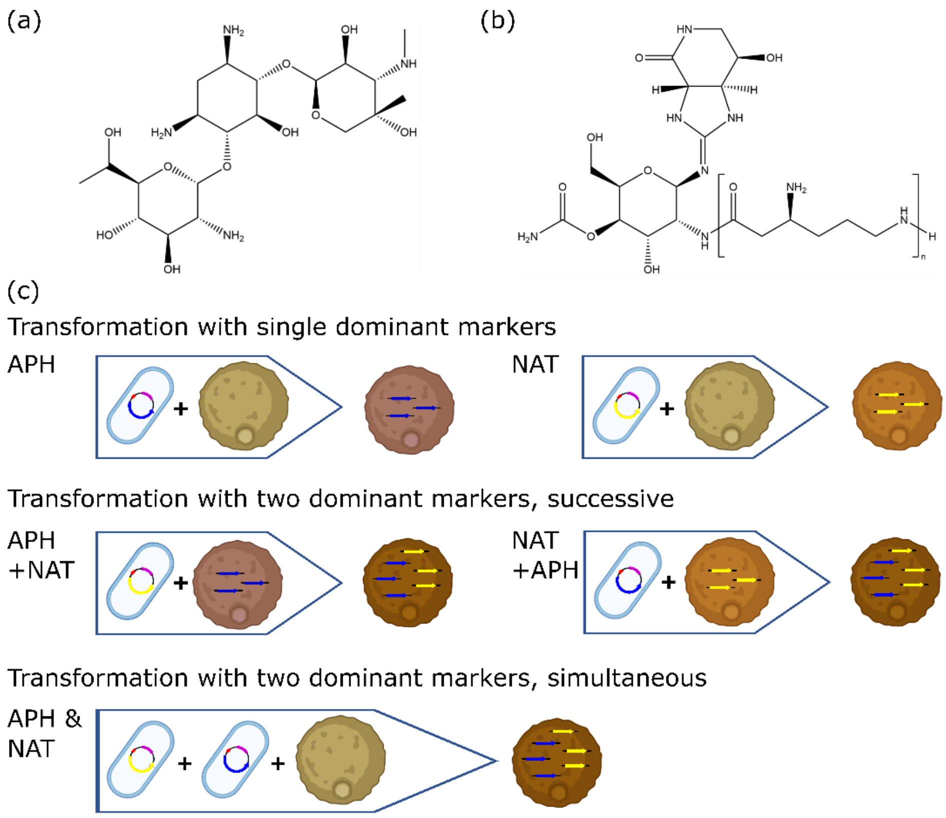

Figure S4. (a) Molecular structure of geneticin G418, created with ChemDraw. (b) Molecular structure of nourseothricin, created with ChemDraw. (c) Transformation strategy to generate single and double transformants with constructs containing the two dominant markers. Parts of the figure were created with BioRender.
